# Confessions of a Quack Doctor

**Published:** 1875-09

**Authors:** 


Confessions of a Quack Doctor.—Mr. G. A. Brine, the pauper in Sherborne Workhouse, whose revelations with regard to the tricks of vagrants have attracted some attention, writes this week to the "Charity Organization Reporter" on the subject of quack doctors and itinerant medicine venders. He says: "I commence with the quack doctors. In the first place I must tell you that I never engaged in the dirty business on my own account. I have been a tool in the hands of others. The first time it was in Yarmouth. A quack, who was lodging in the same 'ken' with me, asked me if I was willing to earn a couple of shillings easily. I replied in the affirmative. This was, to come into the market-place in the afternoon, while he was expatiating on the virtues of his medicines, and purchase half a dozen boxes of the pills, saying that myself and others had derived immense benefit from their use, and that, for the future, I was resolved never to be without them—the money to pay for them having been given me beforehand by the 'doctor.' Well, I carried out my instructions to the letter, and so well pleased the modern Æsculapius, that in the evening he employed me to work for him at a salary of £1 per week, besides traveling expenses. I was now to be initiated into the sublime mystery of compounding the 'medicine,' almost invariably pills.' My duty was to collect the ingredients; and I now solemnly declare that I got them ready made from the sheepfold or the rabbit-warren. Those from the sheepfold had to be considerably reduced in size, after which they were coated with finely pulverized sugar and flour, and, after being dried to a proper consistency, were placed in pill-boxes, which are easily obtained, and then held forth to the dolts, who were silly enough to listen to him, as 'American sugar-coated pills,' purely vegetable, and warranted not to contain one particle of mercury, colocynth, or other deleterious poison, so extensively used by regular doctors. These pills are a sovereign remedy for bilious disorders, liver complaints, dyspepsia, or indigestion, the symptoms of which are learnedly described by the 'orator' (which was generally myself), learnt by heart from a medical work by Dr. Buchanan.

"When we were traveling in country villages, there was no 'ill which flesh is heir to' but what my master (blatant ignoramous as he was) would undertake to cure—worms, piles, tusky or itch, gout, rheumatism, ulcers, fits, etc.; but the naked truth is, that he was a greater fool than I; he could not read a paragraph in a newspaper, and could scarcely write his own name. He knew no more about the maladies he professed to cure than a hog; but he possessed in an eminent degree that grand, indispensable qualification, any amount of cheek, and his takings, on an average, were £10 a week."—*Brit. Med. Jour.*

